# A Quantitative Observational Study of Physician Influence on Hospital Costs

**DOI:** 10.1177/0046958018800906

**Published:** 2018-09-28

**Authors:** Herbert Wong, Zeynal Karaca, Teresa B. Gibson

**Affiliations:** 1 U.S. Department of Health and Human Services, Agency for Healthcare Research and Quality, Rockville, MD, USA; 2 IBM Watson Health, Ann Arbor, MI, USA

**Keywords:** physician practice styles, physician influence, costs, hospital costs, physician characteristics, physicians, inpatients, decision-making, observational study, regression analysis

## Abstract

Physicians serve as the nexus of treatment decision-making in hospitalized patients; however, little empirical evidence describes the influence of individual physicians on hospital costs. In this study, we examine the extent to which hospital costs vary across physicians and physician characteristics. We used all-payer data from 2 states representing 15 237 physicians and 2.5 million hospital visits. Regression analysis and propensity score matching were used to understand the role of observable provider characteristics on hospital costs controlling for patient demographics, socioeconomic characteristics, clinical risk, and hospital characteristics. We used hierarchical models to estimate the amount of variation attributable to physicians. We found that the average cost of hospital inpatient stays registered to female physicians was consistently lower across all empirical specifications when compared with male physicians. We also found a negative association between physicians’ years of experience and the average costs. The average cost of hospital inpatient stays registered to foreign-trained physicians was lower than US-trained physicians. We observed sizable variation in average costs of hospital inpatient stays across medical specialties. In addition, we used hierarchical methods and estimated the amount of remaining variation attributable to physicians and found that it was nonnegligible (intraclass correlation coefficient [ICC]: 0.33 in the full sample). Historically, most physicians have been reimbursed separately from hospitals, and our study shows that physicians play a role in influencing hospital costs. Future policies and practices should acknowledge these important dependencies. This study lends further support for alignment of physician and hospital incentives to control costs and improve outcomes.

**What do we already know about this topic?**
Specific physician characteristics influence a physician’s practice style as well as health care cost, delivery of care and outcomes.
**How does your research contribute to the field?**
Our research expands the current literature by performing an all-payer (vs single payer) analysis, using hierarchical models to estimate the amount of variation attributable to individual physicians, and partitioning the variation in hospital costs to understand the extent of influence attributable to physicians.
**What are your research’s implications toward theory, practice, or policy?**
We found substantial variation in hospital costs with observable physician characteristics, lending further support for payment and organizational models that align physician and hospital incentives that seek to control costs and improve outcomes.

## Introduction

It has been well established that health care spending varies with geography.^1-3^ The source of this variation has been often questioned—whether it is arising from area practice patterns, patient health status, patient characteristics, price, and/or individual provider decision making.^3,4^

An Institute of Medicine (IoM) Committee examining geographic variations in Medicare spending convened earlier this decade concluded that individual providers of care had a great deal of influence on spending.^5^ The Committee found post-acute care and inpatient care had the largest amounts of variation in spending, and discovered large variations in provider behavior.^4^ Recommendations from the IoM Committee stated that evidence pointed away from geographic or small area spending signatures and toward health care decision-makers. Similarly, Gottleib and colleagues^6^ performed a study of spending variation controlling for patient demographic characteristics, health status, and prices between regions, and found that price contributed only a small fraction of variation in spending although patients with similar characteristics received different levels of care from providers.

Previous studies have long demonstrated that specific physician characteristics influence a physician’s practice style.^7-10^ Several studies have assessed how well physician characteristics explain the variation in hospital resource use.^1,11-13^ Other researchers profiled physicians by analyzing and comparing the effects of their characteristics on health care cost, delivery, and outcomes.^14-19^ A recent study by Tsugawa and colleagues^20^ demonstrated the existence of physician influence on Part B Medicare spending and the extent of spending attributable to physicians.

Other recent studies have examined the relationship between observable characteristics of physicians and health care spending and outcomes. For example, patients treated by graduates of foreign medical schools had lower mortality but higher Medicare Part B payments than those graduating from US medical schools.^21^ Elderly patients with a female physician had lower mortality and readmission rates than male physicians.^22^ In a separate study, no clear pattern was found between patient mortality and physician age for elderly patients, but patients with an older physician had higher Medicare Part B payments.^23^ Also, Southern and colleagues^24^ found that tenure in practice was positively associated with higher risk of mortality and longer lengths of stay in a local hospital system.

In this study, we use all-payer inpatient data from 2 states, Arizona and Florida, to analyze and quantify the extent of physician influence on inpatient hospital costs other than professional services. Hospital care accounts for 32% of national health care expenditures and is the largest expense category in 2015.^25^ In addition, physicians are responsible for selecting the course of care provision and treatment, thereby influencing hospital costs of care.

Our research has 2 aims. First, we describe the relationship between hospital costs and observable characteristics of physicians including physician gender and foreign medical school graduation while controlling for patient demographics, socioeconomic characteristics, clinical risk, and hospital characteristics, although cost in this analysis cannot be distinguished between patients and payers. Second, we measure the fraction of variation in costs of hospital inpatient visits due to individual physicians, controlling for observable physician characteristics, patient demographics, socioeconomic characteristics, clinical risk, and hospital characteristics.

This article complements and expands upon the existing empirical literature in several important ways. First, our data are all-payer and do not limit the analysis to a specific payer group or patient group. This extends the previous literature as most recent studies have focused on the physician role in Part B spending variation in large Medicare samples^20-23^ or within small, local samples.^24^ Most physicians have a mix of patients covered by Medicare, Medicaid, private payers, and the uninsured, and we seek to understand their role in influencing hospital costs across all-payer groups. In addition, we use hierarchical models to estimate the amount of variation attributable to individual physicians, controlling for patient demographics, socioeconomic characteristics, clinical risk, and hospital characteristics, allowing us to partition the variation in hospital costs and to understand the extent of influence attributable to physicians. Finally, we use regression analysis and propensity score matching to further understand the role of providers on hospital costs.

## Methods

### Data

We used the Healthcare Cost and Utilization Project (HCUP) 2008 State Inpatient Databases (SID) for Arizona and Florida. These HCUP SID files include all inpatient hospitalizations for nearly all acute care nonfederal hospitals in the subject states. The SID provide detailed diagnoses and procedures, total charges, and patient demographics including gender, age, race, and expected payment source (ie, Medicare, Medicaid, private insurance, other insurance, and self-pay). Physician characteristic information (eg, specialty, year of graduation from medical school, and the name of the medical school) was obtained from the Arizona Board of Medical Examiners and the Florida Department of Health. With permissions from all data partners, information was linked to the Arizona SID using both physician license number and physician name, and to the Florida SID using physician license numbers as the Florida SID do not provide physician name.^i^ The physician represented the surgeon (operating physician), if a surgery was performed, otherwise, the attending physician who is responsible for overall care from admission to discharge. In addition, supplemental hospital characteristic and area characteristic information were obtained, respectively, from the American Hospital Association (AHA) and Area Resource Files. The total number of hospital inpatient visits during 2008 in Arizona and Florida was about 3.31 million, and about 5% were missing physician identifiers. We successfully linked 2.53 million of these visits to physician licensure databases and AHA hospital survey data. All investigators signed a Data Use Agreement. Because HCUP does not involve human subjects, institutional review board approval was not required for this study.

Our key covariates of interest were the physician’s gender, years of experience, board certified specialties, and whether they graduated from a medical school outside of the United States. We calculated years of experience as the difference between 2008 and the year the physician graduated from medical school. We created a series of dummy variables to represent the physician’s specialties of surgery, internal medicine, obstetrics and gynecology, neurology, psychiatry, pediatrics, cardiology, family medicine and general practitioners, and urology. The effect of being foreign-trained was examined by including a separate dummy variable for physicians if they graduated from a medical school outside of the United States. While physicians’ names and the name of their medical school are included in physicians’ licensure databases, physicians’ gender and the location of their medical schools are not readily available. We obtained these from various data sources and online search engines including http://doctor.webmd.com, http://www.aamc.org, http://www.babynames.com, and http://www.google.com. For physician gender, we followed a systematic assignment process requiring matching information from at least 2 independent data sources. Complete information regarding major physician characteristics was obtained for 2.53 million discharges studied in this study.

Hospital costs represent the underlying expenses to produce the hospital services. Since hospitals differ in their markup from costs to charges, we first reduced the charge for each case based on the hospital’s all-payer, inpatient cost-to-charge ratio.^ii^ We applied hospital-specific all-payer cost-to-charge ratios, and replaced all-payer cost-to-charge ratios with group-average all-payer inpatient cost-to-charge ratios when hospital-specific all-payer inpatient cost-to-charge ratios were missing. Next, we adjusted these costs with the area wage index^iii^ computed by the Centers for Medicare and Medicaid Services (CMS) to control for price factors beyond the hospital’s control. We also obtained information about hospital characteristics (eg, teaching status and bed size) using the AHA Annual Survey Database.

### Empirical Models

Our study’s empirical models employ a hierarchical framework^26^ to assess the effects of physician characteristics on the costs of hospital inpatient visits; we developed a model that controls for physician characteristics, patient demographics, socioeconomic characteristics, clinical risk, and hospital characteristics.

We reassessed the impacts of physician characteristics on costs of hospital inpatient visits using multilevel regression analysis where hospital inpatient visits were clustered by physician.

Our empirical model takes the following general form:

(1) $$\begin{array}{c} {\mathrm{LogCost}}_{ij}=\beta_{01j}\;+\;\beta_{11}{\mathrm{demographic}}_{i}+\;\beta_{21}\;{\mathrm{socioecon}}_{i}\\ +\;\beta_{31}\;{\mathrm{risk}}_{i}+\;\;\beta_{41}\;{\mathrm{severity}}_{i}\\ +\beta_{51}\;{\mathrm{hospital}}_{i}+\xi_{ij}, \end{array}$$

where variation in the intercept is predicted at level 2 by

(2) $$\beta_{01j}=\beta_{02}+\beta_{12}{\mathrm{physician}}_{j}+\gamma_{j}.$$

Substituting (2) into Equation (1) yields the following single multilevel equation:

(3) $$\begin{array}{c} {\mathrm{LogCost}}_{ij}=\beta_{02}+\beta_{12}{\mathrm{physician}}_{j}+\;\beta_{01j}\\ +\;\beta_{11}{\mathrm{demographic}}_{i}+\;\beta_{21}\;{\mathrm{socioecon}}_{i}\\ +\;\beta_{31}\;{\mathrm{risk}}_{i}+\;\beta_{41}\;{\mathrm{severity}}_{i}\\ +\;\beta_{51}\;{\mathrm{hospital}}_{i}+\;\gamma_{j}+\xi_{ij}, \end{array}$$

where *i* indexes the hospital inpatient visits and *j* indexes the physicians who treated the *i*th visit, and LogCost_*ij*_ is the natural log value of the total hospital inpatient cost associated with the *i*th visit in the *j*th physician unit. Physician_j_ is a vector of physicians’ characteristics that includes physicians’ years of experience measured as the difference between 2008 and their year of graduation from medical school, a set of dummy variables for physicians’ board certified specialties (surgery, internal medicine, obstetrics and gynecology, neurology, psychiatry, pediatrics, cardiology, family medicine and general practitioners, urology), for physicians’ gender, and for physicians who graduated from a medical school outside of the United States. Demographic_i_ is a vector of observable patient demographic characteristics, which include age (in age/10 scale), and dummy variables for race and gender. Socioecon_*i*_ includes a set of county-level dummy variables for income (low, low-medium, medium-high, and high) and for patients’ primary insurance providers (ie, Medicare, Medicaid, private, and other). Risk_*i*_ includes dummy variables for the Elixhauser comorbidity index.^27^ Severity_*i*_ is the high-severity-measure dummy variable (with value 1 for the patient when All Patient Refined Diagnosis Related Groups (APR-DRG) severity index takes a value of 3 or 4).^28^ Hospital_*i*_ includes a set of dummy variables related to hospital characteristics—including teaching status, ownership type, bed size, and state (Arizona or Florida)—that may also represent unmeasured severity of illness for a patient referred to a highly capable institution. Finally, γ_*j*_ represents departures of the *j*th physician from the overall mean that serves to shift the overall regression line representing the population average up or down according to each physician, and ξ_*ij*_ is the level 1 random error. The random components of this model provide information about intraclass correlation coefficients (ICCs), which enables us to understand variation in costs of hospital inpatient visits associated with physicians’ characteristics. Our level 1 predictor variables are dummy variables except for age, which we standardized by dividing by 10. In our case, centering around the grand mean or using raw metric values did not change the direction of estimates. Therefore, we used raw metric values in our regression analysis.^iv^ We present findings overall, and for teaching and nonteaching hospitals, as physician mix and patient complexity may vary between these types of facilities.

### Sensitivity Analysis

We also developed various scenarios to test the robustness of our results. Specifically, we enhanced our model by incorporating level 2 variation not only in intercept but also in slope. Under this model, we assumed that patients had certain preferences in their choice of physician. We ran 3 models with level 2 variations within physicians by these patient characteristics: gender, severity of illness, and gender and severity of illness. Our empirical findings in these 3 models, where both intercepts and slopes varied in level 2, were parallel to our model with level 2 intercept-only variations. For the purpose of clarity, we provide the results for our base model where level 2 variations are only observed through intercepts that represent departures for each physician from the overall mean.

Some researchers claim that there is an implicit relationship between patient gender and physician gender^7,29-32^ or physicians’ practice style and their graduating medical school,^34^ which could introduce some degree of endogeneity into our empirical model as presented above. Although our multilevel model substantially reduces the unobservable endogeneity by clustering patients across physicians, we employed propensity score matching techniques^33^ to address the potential endogeneity issues when estimating the impact of physicians’ practice style on hospital inpatient costs. We employed the propensity score nearest neighbor (NN) matching without replacement method to create subsamples of physicians based on their observable characteristics. We created our first subsample of physicians by matching female physicians with male physicians based on their observable characteristic of medical specialties, experience, foreign- versus US-trained, state (Arizona or Florida), and whether physicians practiced at both teaching and nonteaching hospitals. Then, we reestimated our multilevel model using hospital inpatient visits registered to these matched cohorts of physicians. The new estimates provide more robust findings regarding the impact of practice styles of female physicians on hospital inpatient costs when compared with their matching male cohorts. Next, we created our second subsample of physicians by matching foreign-trained physicians with US-trained physicians based on their observable characteristics of medical specialties, experience, gender, state, and whether physicians practiced at both teaching and nonteaching hospitals and reestimated our multilevel model.

## Results

The average cost of hospital inpatient visits was $9172 for all visits, $9492 for visits to teaching hospitals, and $8679 for visits to nonteaching hospitals (see Appendix Table A1 for visit characteristics). There were 7993 physicians who worked only at teaching hospitals, 4249 physicians who worked only at nonteaching hospitals, and 2995 physicians who worked in both settings for a total of 15 237 physicians (Table 1). The physicians had an average of 24 years of experience. The proportion of female physicians was 26.5%, and the relative distribution working only at teaching hospitals or nonteaching hospitals, or at both, were comparable. About a third of the physicians graduated from medical schools outside of the United States, and we observed a higher prevalence at nonteaching hospitals when compared with teaching hospitals. We also observed that 16.4% of physicians in our sample were board certified surgeons and 31.7% of physicians had board certification in internal medicine. The percentage of physicians with other board certified specialties was lower: obstetrics and gynecology (8.0), neurology (2.3), psychiatry (1.2), pediatrics (12.7), cardiology (7.0), family medicine and general practitioners (7.3), urology (2.5).

**Table 1.** Profile of Physicians at Hospital Inpatient Settings.

	All physicians	Physicians who work only at teaching hospitals	Physicians who work only at nonteaching hospitals	Physicians who work at both teaching and nonteaching hospitals
Number of physicians (% of total)	15 237 (100%)	7993 (52.5%)	4249 (27.9%)	2995 (19.6%)
Physician characteristics				
Average experience (in years)	24.3	25.0	23.1	23.8
Female	26.5%	27.4%	26.8%	23.7%
Foreign-trained	35.5%	33.5%	37.1%	38.4%
Board certified specialties				
Surgery	16.4%	15.8%	15.2%	19.7%
Internal medicine	31.7	32.1	31.9	30.9
Obstetrics and gynecology	8.0	8.0	8.8	7.2
Neurology	2.3	3.1	1.3	1.7
Psychiatry	1.2	1.8	1.2	0.2
Pediatrics	12.7	13.1	11.2	13.6
Cardiology	7.0	7.5	5.8	7.6
Family medicine and general practitioners	7.3	6.9	9.1	5.8
Urology	2.5	2.2	2.5	3.3
Percentage of visits by high-severity^a^ patients across physician characteristics				
Female	26.2%	25.6%	25.9%	28.3%
Male	28.1	27.2	28.4	29.4
Foreign-trained	24.6%	29.3%	33.5%	32.1%
US-trained	31.3	25.1	28.4	26.2
Board certified specialty				
Surgery	28.1%	29.3%	23.2%	29.7%
Internal medicine	39.3	38.2	41.3	38.7
Obstetrics and gynecology	5.9	7.0	4.2	6.2
Neurology	20.5	20.8	19.8	19.7
Psychiatry	12.2	11.8	11.6	21.1
Pediatrics	10.2	11.8	6.7	11.0
Cardiology	27.7	28.5	26.1	27.5
Family medicine and general practitioners	34.1	33.1	34.5	35.7
Urology	14.2	15.0	13.1	13.7
Average cost of hospital inpatient visit by physician characteristic				
Female	$7482	$7539	$7078	$7903
Male	9746	10 001	8893	10 240
Foreign-trained	$8508	$8316	$8387	$8959
US-trained	9699	10 001	8455	10 431
Surgery	$17 431	$18 353	$15 194	$17 542
Internal medicine	9582	9423	9712	9700
Obstetrics and gynecology	4311	4347	4006	4741
Neurology	16 496	16 286	17 207	16 720
Psychiatry	4724	4875	4235	4308
Pediatrics	4217	4893	2373	4960
Cardiology	14 714	13 732	16 455	8314
Family medicine and general practitioners	8000	7804	8086	8750
Urology	9597	10 538	8578	8959

*Note.* Data include all hospital inpatient stays incurred during 2008 in Arizona and Florida. We excluded all records associated with physicians with 12 or fewer observations during 2008, which is about 1% of entire sample.

a Severity score of 3 or 4 on the APR-DRG severity index (a product of 3M Health Information System).

Table 1 also presents the average cost per hospital inpatient visit by physician characteristics. The average cost of hospital inpatient visits for patients visiting female physicians was $2264 lower when compared with costs for patients visiting male physicians. This difference was larger in teaching hospitals when compared with nonteaching hospitals. Similarly, we observed the average cost per hospital visit treated by foreign-trained physicians was $1191 less when compared with physicians who graduated from a medical college in the United States. Although we observed a larger difference in average hospital inpatient costs between foreign-trained and US-trained physicians who work only at teaching hospitals, there was only about $64 difference for physicians working only at nonteaching hospitals. We found sizable variation in the average cost of a hospital inpatient visit across physicians’ specialties. Patients treated by physicians with specialties in surgery, neurology, and cardiology had relatively higher average costs per hospital visit, which were $17 431, $16 496, and $14 714, respectively.

We also documented the distribution of patients’ severity of illness by physician characteristics. The results presented in Table 1 show that the percentage with high severity of illness was higher for male patients than for female patients regardless of the hospital setting. We also observed that foreign-trained physicians had a relatively higher share of high-severity patients at teaching hospitals when compared with nonteaching hospitals. Finally, we found that the relative share of high-severity patients was greater for physicians with specialties in internal medicine or family medicine and general practitioners working at nonteaching hospitals when compared with physicians with the same specialties working at teaching hospitals. However, for most of the remaining physicians working only at teaching hospitals, we observed a higher share of patients with high severity of illness when compared with physicians working only at nonteaching hospitals.

### Regression Results

Linear regression results presented in column 1 of Table 2 show that the average cost of hospital inpatient visits for patients visiting female physicians was 0.1% lower than male physicians and was 0.5% lower for patients visiting foreign-trained physicians versus US-trained physicians. Each additional year of experience was associated with 4.3% lower costs. We also observed sizable variation in average costs of hospital inpatient visits across medical specialties where surgeons and cardiologists were associated with the highest average cost and pediatricians and psychiatrists were associated with the lowest average cost per hospital inpatient visit. The regression results based on hospital inpatient visits to teaching hospitals were parallel to our main results for all key covariates (Table 2, column 2). We also found similar results for nonteaching hospitals (Table 2, column 3).

Columns 4 to 6 of Table 2 present the results of multilevel regressions estimated separately for hospital inpatient visits to all hospitals, to teaching hospitals, and to nonteaching hospitals to assess the robustness of our earlier results derived from single-level linear regression. The average cost of hospital inpatient visits for patients visiting female physicians was 11% lower than male physicians and was 3.6% lower for patients visiting foreign-trained physicians versus US-trained physicians. Each additional year of experience was associated with 0.10% lower costs. Similar to our earlier results, we found substantial variation in costs of hospital inpatient visits across medical specialties. The multilevel regression results based on inpatient visits to teaching hospitals and nonteaching hospitals retained the same sign and statistical significance, which enhanced the validity and robustness of our results, specifically how physician characteristics impact the cost per hospital inpatient visits.^v^

**Table 2.** Estimated Effects of Physician Characteristics on Log Inpatient Cost Per Visit.

	Linear regression model			Multilevel regression model		
	All visits (1)	Teaching hospitals (2)	Nonteaching hospitals (3)	All visits (4)	Teaching hospitals (5)	Nonteaching hospitals (6)
Physician characteristics						
Experience (in years)	−0.044***	−0.001***	−0.002***	−0.001**	−0.001**	−0.002***
	(0.001)	(0.000)	(0.000)	(0.000)	(0.000)	(0.000)
Female	−0.001***	−0.057***	−0.032***	−0.117***	−0.137***	−0.097***
	(0.000)	(0.002)	(0.002)	(0.010)	(0.013)	(0.014)
Foreign-trained	−0.005***	−0.005***	−0.007***	−0.037***	−0.032***	−0.034***
	(0.001)	(0.002)	(0.002)	(0.009)	(0.012)	(0.013)
Board certified specialties						
Surgery	0.804***	0.806***	0.789***	0.722***	0.732***	0.712***
	(0.002)	(0.002)	(0.003)	(0.013)	(0.017)	(0.020)
Internal medicine	0.035***	0.023***	0.061***	0.046***	0.037**	0.076***
	(0.001)	(0.002)	(0.002)	(0.012)	(0.014)	(0.017)
Obstetrics and gynecology	0.087***	0.074***	0.102***	−0.089***	−0.061***	−0.113***
	(0.002)	(0.003)	(0.003)	(0.017)	(0.022)	(0.025)
Neurology	0.346***	0.320***	0.383***	0.335***	0.308***	0.378***
	(0.004)	(0.005)	(0.008)	(0.028)	(0.032)	(0.052)
Psychiatry	−0.334***	−0.321***	−0.388***	−0.331***	−0.280***	−0.530***
	(0.004)	(0.005)	(0.008)	(0.038)	(0.045)	(0.070)
Pediatrics	−0.618***	−0.577***	−0.690***	−1.067***	−0.989***	−1.272***
	(0.002)	(0.003)	(0.003)	(0.014)	(0.018)	(0.021)
Cardiology	0.523***	0.462***	0.643***	0.537***	0.507***	0.566***
	(0.003)	(0.004)	(0.005)	(0.018)	(0.022)	(0.026)
Family medicine and general practitioners	−0.044***	−0.080***	0.007***	−0.099***	−0.139***	−0.036
	(0.002)	(0.003)	(0.003)	(0.017)	(0.022)	(0.024)
Urology	0.415***	0.448***	0.358***	0.366***	0.389***	0.300***
	(0.006)	(0.008)	(0.009)	(0.028)	(0.036)	(0.039)
*R* ^2^	0.477	0.462	0.509			
Total inpatient visits	2 396 882	1 457 972	938 910	2 396 882	1 457 972	938 910
Variance (level 1 estimate)				0.466	0.489	0.419
Variance (level 2 estimate)				0.257	0.280	0.241

*Note.* Data include all hospital inpatient stays incurred during 2008 in Arizona and Florida. We excluded all records associated with physicians with 12 or fewer observations during 2008, which is about 1% of the entire sample. All regression models include patient’s primary payers, median household income for residences in patient’s ZIP Code, and the Elixhauser comorbidity index. Level 1 is visit level and level 2 is physician level. Percent impact is calculated as (exp(coefficient) – 1) × 100. Standard errors are in parentheses.

* *P* < .10. **P < .05. ***P < .01.

### Sensitivity Analysis

Table 2 presents the estimates separately for 2 cohorts of physicians where the first cohort includes equal numbers of male and female physicians with a similar distribution of other characteristics, and the second cohort includes equal numbers of foreign-trained and US-trained physicians with a similar distribution of other characteristics (see matching results in Appendix Table A2). The estimated coefficients on key physician characteristics are highly statistically significant and have the same direction as our earlier results. In our female-male matched cohort, the regression results show that the hospital inpatient costs registered to female physicians are 10.8% lower when compared with hospital inpatient visits registered to male physicians. Similarly, the estimated effect of foreign-trained physicians on hospital inpatient costs is 3.8% lower in our second cohort where each foreign-trained physician is matched with a US-trained physician. The coefficients on physicians’ experience and medical specialties remain statistically significant and parallel to our earlier findings in the hierarchical models.

The multilevel regression results presented in Tables 2 and 3 also enable us to empirically measure the average correlation of patients registered to the same physicians. ICC, which is calculated by dividing the level 1 variance by the sum of the level 1 and level 2 variations, describes how strongly hospital inpatient visits registered to the same physicians are correlated with each other. In general, if ICC approaches to value zero, then one might chose to ignore multilevel estimation models and analyze the data in standard ways. On the contrary, if the ICC approaches the value one, there is no variation among patients registered to same physicians, so one might aggregate the data at the physician level and run a single-level linear regression model on aggregated data. For our case, the ICC values ranged from 0.329 (0.241 / [0.241 + 0.419]) (nonteaching hospitals) to 0.364 (teaching hospitals) (Table 2) before matching and 0.605 (female models) to 0.643 (0.481 / [0.481 + 0.267]) (foreign-trained physician models) (Table 3) after matching which indicates modest to sizable variation among visits registered to the same physician. The ICC range of our multilevel model also empirically validates our discussion around the necessity of using a multilevel model rather than single-level linear regression model.

**Table 3.** Estimated Effects of Physician Characteristics on Log Inpatient Spending Per Visit.

	Propensity score nearest neighbor matched for	
	Female physicians^a^	Foreign-trained physicians^b^
Physician characteristics		
Experience (in years)	−0.001*	−0.001**
	(0.001)	(0.000)
Female	−0.114***	−0.111***
	(0.013)	(0.012)
Foreign trained	−0.005	−0.039***
	(0.014)	(0.010)
Board certified specialties		
Surgery	0.798***	0.736***
	(0.028)	(0.021)
Internal medicine	0.106***	0.048***
	(0.017)	(0.013)
Obstetrics and gynecology	−0.035	−0.051*
	(0.023)	(0.031)
Neurology	0.422***	0.311***
	(0.055)	(0.041)
Psychiatry	−0.175***	−0.346***
	(0.066)	(0.041)
Pediatrics	−1.010***	−1.149***
	(0.018)	(0.017)
Cardiology	0.618***	0.523***
	(0.037)	(0.020)
Family medicine and general practitioners	−0.056**	−0.066***
	(0.026)	(0.021)
Urology	0.469***	0.396***
	(0.067)	(0.053)
Total number of physicians	7956	10 596
Total inpatient visits	1 341 138	1 792 693
Variance (level 1 estimate)	0.311	0.267
Variance (level 2 estimate)	0.476	0.481

*Note.* Level 1 is visit level and level 2 is physician level. Percent impact is calculated as (exp(coefficient) – 1) × 100. NN = nearest neighbor. Absolute values of *t*-ratios are in parentheses.

a Data include all hospital inpatient visits registered to physicians associated with at least 12 inpatient hospital visits records during 2008 in Arizona or Florida. Each female physician is matched with a male physician based on the propensity score NN matching without replacement. The regression model controls for patients’ gender, race, age, primary payers, high severity of illness, and Elixhauser comorbidity index. The model also includes dummy variables for hospitals’ teaching status, ownership type, bed size capacity, and median household income for a residence in patients’ ZIP Code.

b Data include all hospital inpatient visits registered to physicians associated with at least 12 inpatient hospital visit records during 2008 in Arizona or Florida. Each physician who graduated from a medical school outside of the United States is matched to a physician who graduated from a medical school located in the United States based on propensity score NN matching without replacement. The regression model controls for patients’ gender, race, age, primary payers, high severity of illness, and Elixhauser comorbidity index. The model also includes dummy variables for hospitals’ teaching status, ownership type, bed size capacity, and median household income for a residence in patients’ ZIP Code.

* *P* < .10. **P < .05. ***P < .01.

## Discussion

In this examination of all-payer data from two states, we found substantial variation in the costs of producing these hospital services with observable physician characteristics such as physician age, gender, foreign training, and physician specialty. We found that the average cost of hospital inpatient stays registered to female physicians was consistently lower across all empirical specifications when compared with the average cost of hospital inpatient stays registered to male physicians. We also found a negative association between physicians’ years of experience and the average costs of hospital inpatient stays. Similarly, the average cost of hospital inpatient stays registered to foreign-trained physicians was significantly lower when compared with the average cost of hospital inpatient stays registered to US-trained physicians. Finally, we observed sizable variation in average costs of hospital inpatient stays across medical specialties where surgeons and cardiologists were generally associated with higher average costs and pediatricians and psychiatrists were generally associated with lower average costs. Further research should investigate the sources of the differences associated with physician characteristics.

Using hierarchical methods and random effects, we estimated the percentage of remaining variation attributable to individual physicians. Using the entire sample, the ICC was approximately 0.35, or one third of the variation was attributable to physicians. Our approach partitions the variation in hospital costs and allows physicians to practice at multiple hospitals. Other studies have employed hospital fixed effects and partitioned the remaining variation in physician costs effectively comparing physicians within the same hospital.^21^ This is an important distinction in approaches and could result in slightly different conclusions based on the variation that is being partitioned (total or net of hospital fixed effects).

Our data are all-payer and focus on the underlying costs of providing care. These differ from reimbursement amounts which may be relatively standardized across hospitals through Diagnosis Related Groups (DRG) payments within payers. Our results confirm that physician behavior is associated with variation in hospital costs other than professional services and this could occur through variations in physician practice styles and treatment decision-making.

Our study is limited to data from 2 large US states, and analysis of physician behavior in other states or countries may differ. This study relies on accurate attribution of individual physicians to hospital discharges. Our study is not experimental, and is observational, revealing a retrospective view of the association between physicians and hospital costs. Physicians were not randomized to patients, so potential endogeneity exists in patient selection of physicians. We attempted to minimize the impact of potential endogeneity in physician gender and foreign-trained physicians by creating matched samples of physicians and found that the ICC increased substantially, exceeding 0.60. This result is likely due to the retention of more similar samples of physicians, where residual variation is lower, and the percentage of variation attributable to physicians is higher.

When compared with recent studies, our findings are consistent with Tsugawa and colleagues^22^ who found that female physicians treating Medicare patients had lower Part B payments. However, while we found that foreign medical graduates had slightly lower hospital costs, Tsugawa and colleagues^21^ also found that foreign medical graduates had slightly higher Part B spending ($47 per discharge). The difference may be in the data used; ours is all-payer and focuses on hospital costs, and Tsugawa and colleagues^21^ analyze Medicare enrollees and Medicare Part B payments as well as a methodological difference. Tsugawa and colleagues employ hospital fixed effects which compares physicians practicing at the same hospital.

Historically, physician and hospitals have been reimbursed via separate mechanisms, and our results quantify the physician role in the provision of care in hospital facilities. Our study lends support to the interconnected relationship between physicians and facilities in providing care to patients. Future policies, practices, and training processes for hospital administrators and physicians should acknowledge and address these important dependencies.

This study predates large systemic changes to align incentives of physicians and hospitals including some types of Alternative Payment Models and Accountable Care Organizations, and allows a window into physician influence on hospital costs prior to the expansion of these initiatives. At the time of this study, physicians generally had fewer incentives to control hospital costs. As aligned incentives expand, repeating this analysis will be important to understand trends in cost variation and physician influence. Our study also lends further support for payment and organizational models that align physician and hospital incentives that seek to control costs and improve outcomes.

## Appendix

**Table A1.** Profile of Hospital Inpatient Visits.

	All visits	Teaching hospitals	Nonteaching hospitals
Total number of visits (% of total)	2 538 260 (100.0%)	1 541 290 (60.7%)	996 970 (39.3%)
Average cost per visit	$9172.70	$9491.80	$8679.30
Patient characteristics			
Age			
Below 15	13.7%	13.5%	13.8%
15-24	6.9	7.0	6.9
25-44	17.9	18.4	17.1
45-64	23.9	24.6	22.7
65-74	14.4	13.9	15.1
Above 74	23.3	22.5	24.4
Female	56.9%	56.3%	57.8%
Race			
White	66.8%	63.3%	72.2%
Black	13.1	15.8	8.9
Hispanic	14.5	14.1	15.1
Asian	0.9	0.9	1.0
Native	1.0	0.8	1.4
Other	3.7	5.2	1.4
Insurance coverage			
Medicare	41.0%	40.5%	41.7%
Medicaid	19.2	20.0	17.8
Private	29.0	27.5	31.3
Other	3.9	4.3	3.3
Uninsured	6.9	8.7	5.9
High severity^a^	27.6%	27.5%	27.8%
Number of different chronic conditions			
0	37.7%	37.1%	38.6%
1	23.0	22.8	23.4
2	19.6	19.8	19.5
3	11.8	12.1	11.4
4	5.3	5.6	4.9
5 or more	2.5	2.7	2.2
Hospital and area characteristics			
Share of visits in Florida	73.4%	84.5%	56.3%
Large hospitals, bed size ⩾300	56.1	63.6	44.6
For-profit hospitals	47.9	47.2	49.1
Low income^b^	32.6	35.3	28.3
Low-medium income	32.1	30.7	34.4
Medium-high income	22.4	22.4	22.3
High income	12.9	11.6	15.0

*Note.* Data include all hospital inpatient stays incurred during 2008 in Arizona and Florida. We excluded all records associated with physicians with 12 or fewer observations during 2008, which was about 1% of the entire sample.

a Severity score of 3 or 4 on the APR-DRG severity index (a product of 3M Health Information System)

b Median household income of residences in patient’s ZIP Code. Further details are available at http://www.hcup-us.ahrq.gov/db/vars/zipinc_qrtl/nisnote.jsp.

### Analytic Framework for Hierarchical Models

Following existing studies on multilevel models (Bryk and Raudenbush 1992, Rice and Jones 1997, Carey 2000, Diez-Roux 2000), a basic formal 2-level model is presented, with a single level 1 predictor and a single level 2 predictor with the intercept modeled to vary randomly at level 2. The level 1 model takes the form

(1) $$Y_{ij}=\beta_{01j}+\;\;\beta_{11}X_{i}+\;\xi_{ij}.$$

Response variable *Y* represents hospital inpatient cost per visit, *X* is a predictor that varies with hospital inpatient visits, and subscripts *i* and *j* reference hospital inpatient visits and physicians, respectively. Residual $\xi_{ij}$ is the random error for the *i*th hospital inpatient visit in the *j*th physician unit. At level 2, variation in the intercept is predicted by

(2) $$\beta_{01j}=\beta_{02}+\;\beta_{12}P_{j}+\;\gamma_{j}\;.$$

The terms β_02_ represent fixed elements and β_12_, the coefficients on *P_j_*, varies for each physician. The terms γ_*j*_ are the random error components and along with ξ_*ij*_ are assumed to be normally distributed with zero mean. Furthermore,

$$\mathrm{var}\left(\xi_{ij}\right)=\sigma_{\xi },\;\;\mathrm{var}\left(\gamma_{j}\right)=\sigma_{\gamma_{j}},\;\;\mathrm{cov}\left(\xi_{ij},\;\gamma_{j}\right)=0.$$

Substituting (2) into Equation (1) yields the single 2-level multilevel equation:

(3) $$Y_{ij}=\;\beta_{02}+\;\beta_{11}X_{i}+\;\beta_{12}P_{j}+\left(\gamma_{j}+\;\xi_{ij}\right).$$

The first 3 terms on the right-hand side make up the deterministic part of the model. The 2 terms in parentheses comprise the stochastic or residual portion, which, in this example, contains 2 random variables. Components $\gamma_{j}$ represent departures of the *j*th physician from the overall mean; $\xi_{ij}$ is the hospital-inpatient-visit-level random error. Equation (3) requires the estimation of 3 fixed coefficients, 2 variances, and one covariance component. The presence of more than one residual term distinguishes this model from standard regression models. It is straightforward to enhance this model with more predictors and higher levels.

We present the descriptive characteristics for the 2 separate subsamples of physicians obtained from the propensity score nearest neighbor (NN) matching without replacement method as explained earlier. The descriptive results presented in Table A2 shows that the distribution of physician characteristics in the matching cohorts are very similar. For example, 3978 female physicians were individually matched with 3978 male physicians based on their observable characteristics. In this sample, the relative distribution of medical specialties and mean value for experience among female physicians were very similar to those of their male physician counterparts. Similarly, Table A2 shows that 5298 foreign-trained physicians were matched with 5298 US-trained physicians. The relative distributions of medical specialties, gender, and mean value for experience for foreign-trained physicians were very close to those statistics among the US-trained physicians.

**Table A2.** Profile of Physicians Matched Through Propensity Score NN Matching Without Replacement Method.

	Female physicians^a^		Foreign-trained physicians^b^	
	Female	Male	Foreign	United States
Number of matched physicians	3978	3978	5298	5298
Physician characteristics				
Average experience (in years)	20.6	21	25.9	25.9
Female	50.0%	50.0%	27.2%	24.9%
Foreign	36.2	39.7	50.0	50.0
Board certified specialties				
Surgery	6.6%	6.7%	8.1%	8.1%
Internal medicine	28.3	29.6	42.6	42.3
Obstetrics and gynecology	11.7	10.3	3.2	2.9
Neurology	1.4	1.5	1.7	1.6
Psychiatry	1.2	0.7	1.7	1.5
Pediatrics	21.7	20.0	12.1	13.0
Cardiology	3.3	3.2	7.2	8.1
Family medicine and general practitioners	7.6	7.4	6.8	8.2
Urology	0.9	1.0	1.1	0.9

*Note.* NN = nearest neighbor.

a Data include all physicians registered to at least 12 inpatient hospital stay records during 2008 in Arizona or Florida. Each female physician is matched with a male physician based on propensity score NN matching without replacement.

b Data include all physicians registered to least 12 inpatient hospital stay records during 2008 in Arizona or Florida. Each physician who graduated from a medical school outside of the United States is matched to a physician who graduated from a medical school located in the United States based on propensity score NN matching without replacement.

## Notes

i. Arizona Department of Health and Florida Agency for Health Care Administration granted special permission to access physician identifiers used by the research team

ii. The methodology uses the hospital’s accounting report covering all patients submitted to Centers for Medicare and Medicaid Services (CMS) and is described in user guides at http://www.hcup-us.ahrq.gov/db/state/costtocharge.jsp (accessed October 10, 2012).

iii. Costs throughout this article are inflation-adjusted. The methodology is described in user guides at http://www.hcup-us.ahrq.gov/db/state/costtocharge.jsp (accessed October 10, 2012).

iv. We also used grand mean centering for all level 1 variables (except age, which was scaled as age/10), and we found the direction and significance of results remained same.

v. Some researchers may suggest further clustering instead of estimating 2-level multilevel regression model separately using patients’ discharge data from teaching hospitals and nonteaching hospitals. We added further clustering by hospital’s teaching status and regression results for 3-level multilevel regression model were parallel to our earlier 2-level multilevel regression results.
